# A novel music-based therapeutic approach: the Therapeutic Music Listening

**DOI:** 10.3389/fnhum.2023.1204593

**Published:** 2023-07-14

**Authors:** Alfredo Raglio

**Affiliations:** Istituti Clinici Scientifici Maugeri IRCCS, Pavia, Italy

**Keywords:** music listening, Therapeutic Music Listening, music therapy, evidence-based approaches, clinical and psychological symptoms

## Abstract

The therapeutic use of music is frequently based on active interventions that directly involve the patient through a sonorous-music interaction with the music therapist. In contrast, approaches based on musical listening are characterized by a relationship aimed at promoting an introspective work and processing of one’s emotional experiences. Increasingly, the scientific literature has shown how even listening to music related to the patient’s personal tastes (preferred music listening) and by-passing the direct relationship with the patient, can produce therapeutic effects in different clinical settings. However, in many cases, a clear therapeutic rationale and specific application protocols are still lacking. The paper introduces a novel approach based on music listening: the Therapeutic Music Listening. This approach integrates the subjective component of listening (patient’s musical tastes) and structural and parametric characteristics of the music in relation to the therapeutic aims. The article defines theoretical-applicative bases as well as therapeutic and research perspectives of this music listening-based intervention.

## 1. Introduction

A careful review of the literature related to the various applications of music in clinical settings, has identified several types of interventions. Music therapy interventions can be divided into relational or rehabilitative approaches, individualized music listening (based on songs chosen by the patient/client with the possible support of a music therapist) or proposed by a therapist (experimenter-selected, based on songs deemed appropriate to achieve the therapeutic aim). Further interventions are based on a generic use of musical activities (making and/or listening to music without specific therapeutic-rehabilitative aims) (Raglio and Oasi, 2015). There is a high level of heterogeneity among music interventions in clinical settings in terms of theoretical assumptions, techniques, intervention protocols, and assessment methods. Additionally, the content of the music intervention is often unclear, and the therapeutic rationale is not always clear and thorough. It is important to distinguish between music therapy interventions with a relational purpose and those with a rehabilitation-related purpose. Relational music therapy approaches constitute the “core” of music therapy (Gold et al., 2009) and include active and receptive techniques. In the active approach, the interaction is through musical instruments, singing, movement and/or composition-improvisation activities, which are used as expressive and communicative vehicles. In the receptive approach the patient, through musical listening and subsequent verbal interactions, engages in an introspective activity that involves his or her emotional experiences. In music therapy practice, music listening plays an important role: a recent report by the World Federation of Music Therapy (Kern and Tague, 2017) reports that this approach characterizes about 47% of music therapy interventions. Receptive approaches distinguish those of a relational nature based on psychologically grounded models, such as the Bonny Method of Guided Imagery and Music (Bonny, 2001; Frohne-Hagemann et al., 2015; Mc Ferran and Grocke, 2022), from music listening approaches independent of specific relational references. It is precisely the latter type of listening that can be defined like self or experimenter-selected music listening. These interventions, in which music is the main therapeutic factor in the absence of a specific relational process that is contextual to music listening, has interesting therapeutic potential in many clinical and preventive settings (Raglio, 2020). However, in many cases, a clear therapeutic rationale and specific application protocols are still lacking. This paper presents an approach referred to here defined as Therapeutic Music Listening (TML), based on functional music listening, which is aimed at reducing/alleviating transient or structured symptoms (e.g., anxiety, stress, pain, etc.) or increasing wellbeing conditions. TML consists of listening to individualized playlists created by a music therapist based on anamnestic and personal data about the user and scientific notions, considering therapeutic aims. This paper proposes the systematization of TML by attempting to define its theoretical assumptions, application, and verification methods. TML definition, aims and mechanisms are summarized in Supplementary Figure 1.

## 2. Theoretical background

Listening to music is connected to the sensory processing of the acoustic characteristics of the musical message by the auditory system. The central nervous system has two auditory pathways: the pathway from the internal ear reaching the auditory cortex and another pathway going to the reticular system, with connections to the limbic and autonomic nervous systems (Boso et al., 2006). This results in an important and extensive involvement of cortical and subcortical areas. Music, and especially music listening, also produces significant effects at the neurochemical level. The neuroendocrine system (hypothalamic-pituitary-adrenal axis) is involved in these processes because of the secretion of corticosteroids, which play an important role in stress management. The sympathoadrenal system mediates the secretion of hormones such as catecholamines, adrenaline and noradrenaline (Chanda and Levitin, 2013; Sihvonen et al., 2017; Finn and Fancourt, 2018). Bernardi et al. (2009) observed how variations in rhythmic and dynamic features within short excerpts of songs corresponded to changes in observed physiological variables (heart rate, respiratory rate, blood pressure, skin conductance). Therefore, it appears, that there is a correspondence between the responses coming from the autonomic nervous system and music, which could, precisely through these changes in parameters, modulate physiological responses in the music listener. Other effects were observed at the cardiovascular autonomic level (Raglio et al., 2010, 2021a, 2022; Loomba et al., 2012; Koelsch and Jäncke, 2015) modulating cardiac activity with obvious possible implications in various clinical settings (Kulinski et al., 2022). Studies performed with neuroimaging (Koelsch, 2014; Chan and Han, 2022) have also emphasized the fundamental role that the limbic system plays in music information processing. In the limbic system and particularly in the nucleus accumbens, opioids are responsible for stimulating dopaminergic activity and the release of this neurotransmitter in the ventral tegmental area and are, therefore, involved, along with dopamine itself, in mediating the brain’s responses to reward. The demonstration concerning the activation of these systems during music processing has been a key aspect in defining the neural basis of music listening and in justifying the gratification derived from it. Evidence now exists regarding the existence of a mechanism through which listening to gratifying music results in positive and rewarding reactions (Salimpoor et al., 2011; Zatorre, 2018). Music-activated reward structures involved in the limbic system include dopaminergic midbrain nuclei, ventral striatum, nucleus accumbens, amygdala, hippocampus, and prefrontal and orbitofrontal structures (Levitin and Tirovolas, 2009). Musical stimuli would thus activate mechanisms of anticipation of a desired stimulus, mediated mainly by dopamine but also by other neurotransmitters such as serotonin, norepinephrine, and endorphins that could help explain the strong motivation with respect to the frequent need for prolonged music listening. These anticipation mechanisms could be related to information processing in auditory areas and be created by the individual from prior subjective experiences of listening. This would also create a relationship between cognitive processes and perceived pleasure in music listening. The role that music could play in modulating the activity of limbic and paralimbic structures has very important implications for the use of music in the treatment of various disorders, such as psychiatric or neurodegenerative disorders, which are known to have abnormalities in dopaminergic production (Koelsch, 2014).

This summary regarding the rationale behind the therapeutic potential of music listening, outlines a positive picture that justifies its use in numerous clinical and preventive settings. Despite the scientific value that emerges from the literature, there is a clear delineation of the need for further research in this regard to adequately connect the specificity of music listening to clinical and application issues. Therefore, greater dialogue and integration between professionals in the clinical, psychological, and neuroscientific fields and music therapy professionals is recommended. This would strengthen the interventions by making them more coherent and rigorous from a scientific and application perspective.

## 3. Clinical-application areas of music listening

Given the above fundamentals, music listening can be used in many clinical settings, including both music therapy receptive techniques and music medicine approaches. Hospital settings is certainly one of the contexts in which music listening can be widely used, especially as music medicine approach. In addition to logistical reasons (e.g., difficulty of setting up an active music therapy setting) and clinical reasons (type and brevity of hospital stays), music listening can be easily implemented during hospital stays. This is in the presence of transient symptoms that characterize the peri-operative setting, pain medicine, sleep medicine, oncological setting, etc., or more widely where the hospital stay is accompanied by psychological symptoms such as anxiety, stress, depression, etc. Often, pharmacological treatment can lead to various side effects. Potentially, music listening can reduce the need for drugs, improve the psychological condition, and, in some cases, even reduce hospitalization time (Hole et al., 2015; Raglio, 2019). Specific studies report, in various clinical situations, a significant reduction in anxiety associated with music listening (Guétin et al., 2009; Hole et al., 2015) as well as a significant effect in reducing stress (Radstaak et al., 2014; Linnemann et al., 2015; De Witte et al., 2020; Raglio et al., 2020a), perceived pain (Hole et al., 2015; Chai et al., 2020; Lee, 2021) and sleep disturbances (Jespersen et al., 2015; Trahan et al., 2018; Cordi et al., 2019; Dickson and Schubert, 2020; Kuula et al., 2020; Loewy, 2020). Listening to music has also been shown to reduce behavioral disorders (Gerdner, 2000, 2012; Sakamoto et al., 2013; Särkämö et al., 2014; Maseda et al., 2018; Gaviola et al., 2019; Kwak et al., 2020) and improving overall cognitive function (Särkämö et al., 2008). These studies are all characterized by extreme heterogeneity in the musical pieces, application protocols, and outcome measures used. This makes it difficult to relate the type of music listening to the effects produced by it. As a result, a practice must be defined that integrates scientific evidence with knowledge derived from music therapeutic, clinical, and neuroscientific fields.

## 4. The Therapeutic Music Listening approach

The approach is applied from a collection of anamnestic data that includes personal and musical information. The questionnaire that precedes the creation of the initial playlist collects information, allowing the creation of a music listening program that combines some fundamental aspects: (a) the preferences of the user; (b) the musical characteristics (structural and parametric) of musical choices; and (c) the therapeutic needs. The listening program aims to consider not only musical preferences, but also, to focus attention on the structural and parametric component of the musical pieces, according to three categories that are broadly reflected in the therapeutic needs, regardless of the type of pathological condition, by-passing the more subjective component related to personal emotional referrals and music liking. The three categories that the therapist refers to in the choices of musical parameters are: relaxation/de-activation, activation, and distraction (shifting attention away from the relevant condition). It is important to note that these categories refer to conditions that can be applied to specific musical structures/parameters. In no way does this deny the subjectivity of the listener, but rather attempts to connect therapeutic needs with more objective music parameters. It follows that relaxing/de-activating songs can be traced back to a tendentially slow tempo (40–60 bpm), characterized by regularity, predictability and reduced rhythmic, melodic, and harmonic complexity. To determine a de-activating effect, it is also important that the song does not have a high expressive intensity and potential personal reference (absence of lyrics or content leading back to personal memories or experiences). Conversely, potentially activating songs will tend to have opposite characteristics: tempo > 60 bpm with greater parametric and structural irregularities and complexities. Songs with distractive value will need to contain divergent elements, irregularities, and a high number of variations, together with modulable parametric and structural complexity. It is possible to assume a gradualness in defining the above categories. Therefore, it is believed that the TML pathway combines the subjective component with the above parametric and structural characteristics of music (Table 1).

**TABLE 1:** Music categories connected to therapeutic aims and related clinical fields of application.

Music categories connected to therapeutic aims	Description	Clinical fields of application (examples)
Activation/Stimulation	● Moderate-fast Tempo ● Medium-high rhythmic complexity ● Melody with broad range intervals ● Medium to high frequency of harmonic variations/modulations ● Extensive instrumental ensemble ● Partial regularity	● Apathy (e.g., as a symptom of dementia) ● Negative symptoms (e.g., in psychiatric patients) ● Continuous low mood or sadness, having no motivation or interest in things (e.g., in depression as main diagnosis or associated to other illness) ● …
De-activation/Relaxing	● Slow Tempo ● Low rhythmic complexity ● Melody with restrictive range intervals ● Low frequency of harmonic variations/modulations ● Solo instrument/Reduced instrumental ensemble. ● Steady musical development	● Anxiety (as main diagnosis or associated to other illness or medical conditions) (Raglio et al., 2021c) ● Stress (associated to medical or life conditions or in work-related stress) (Raglio et al., 2020a) ● Agitation, Irritability (e.g., as symptoms of dementia or as behavioral disturbances in psychiatric/neurological patients) (Raglio et al., 2015) ● Sleep disorders (in many clinical conditions) ● …
Distractiveness (relaxing or activating)	● Presence of frequent musical variations/modulations that capture the listener’s attention (i.e., pitch, tempo, timbre, rhythmic changes, harmonic modulations, etc.) counteracting the symptoms	● Pain (chronic and acute condition) (Raglio et al., 2023) ● Obsessive compulsive disorder ● Attention disorders ● …

A music therapist relies heavily on anamnesis, music and music therapy skills, and data from the literature (when available).

Therapeutic Musical Listening thus constitutes an approach that considers the need to propose listening programs based on the patient’s musical background emphasizing, at the same time, the specificity of musical features.

This makes it possible to create a listening program that allows, through the impact of music on the individual, an entrainment mechanism aimed at regulating and modulating psycho-physiological parameters dynamically, reflecting the patient’s needs.

The modulation and regulation of stimuli (through the choice of specific parameters and the periodic feedback provided during the therapeutic course) allows for a tailored intervention to be proposed while respecting the subjective musical tastes of the client/patient. Musical parameters and structures can reflect and modulate psychophysical needs in a rewarding and predictable context (reward effect, Zatorre, 2018).

The procedure regarding the implementation of TML-based interventions (Table 2) will turn out to be as follows:

**TABLE 2:** Phases of the Therapeutic Music Listening intervention.

1. Anamnesis and personal data collection (Questionnaire 1)
2. Identification of therapeutic aims and assessment (clinical scales and subjective evaluations)
3. Definition of therapeutic objectives and related music categories: a. Activation/Stimulation b. De-activation/Relaxing c. Distractiveness
4. Creation of playlist based on music categories connected to the therapeutic aim and definition of a specific music design (musical development)
5. Immediate feedback (Questionnaire 2)
6. Delivery of playlist, modes, and timing of administration (definition)
7. Daily listening supported by caregiver (if needed)
8. Weekly feedback (Questionnaire 2)
9. Confirmation or creation of a new playlist based on the results found and the evolution of the therapeutic treatment
10. Final assessment (after intervention): subjective (Questionnaire 2), clinical scales (depending on specific therapeutic aims)

Avoid, whenever possible (especially for de-activating/relaxing playlists), songs containing lyrics that may shift the listener’s attention to the meaning of the lyrics.

1. Administration of Questionnaire 1 (personal/clinical data and musical anamnesis) (see Supplementary data).
2. Identification of therapeutic aims and assessment (clinical scales and subjective evaluations).
3. Selection of the most appropriate reference categories (activation/stimulation, de-activation/relaxation, and distraction) based on the identified therapeutic aims, and definition of music “design” that adequately modulates the trend of music parameters.
4. Creation of the individualized listening program (20–40 min).
5. Enjoyment test (post- listening evaluation of the impact on the user, Questionnaire 2) (see Supplementary data).
6. Delivery of individualized listening program (definition of listening mode and timing).
7. Daily listening by patient/client according to agreed modalities.
8. Periodical feedback (interview, preferably weekly, Questionnaire 2).
9. Confirmation or creation of a new individualized listening program based on the results found and the evolution of the therapeutic treatment.
10. Clinical evaluation and subjective assessment (Questionnaire 2) at the end of treatment.

This application system ensures the possibility of creating and monitoring a specific listening program that can be varied and modulated based on what emerges during the treatment. In the case of the patient’s lack of autonomy, the support of health care staff is of paramount importance in facilitating the patient’s access to the program. Devices and modes of listening (e.g., place, listening through headphones or external speakers, etc.) may vary in relation to specific needs or the clinical or logistical features that connote therapeutic treatment. Clinical experience suggests music listening sessions of 20–40 min in duration (preferably with headphones). The time limit often coincides with possible attention span and patient/client availability. The frequency of music listening (daily or, more rarely, multi-daily) should be assessed based on the need and clinical characteristics of the user.

## 5. Discussion

### 5.1. What is new in the Therapeutic Music Listening?

Compared with other receptive music therapy techniques, such as the Bonny Method of Guided Imagery and Music (Bonny, 2001; Frohne-Hagemann et al., 2015), TML does not propose an introspective intervention guided by the music therapist. Rather, the music therapist builds a hypothesis of therapeutic intervention based on the potential effects of music and its parameters in relation to the therapeutic need, modulating and varying the proposals over time based on periodic feedback given by the patient.

This process integrates aspects of perception and the subjective effects of music (the impact of music on the patient) and aspects given using specific musical parameters chosen to achieve specific effects (the choices of the music therapist).

As anticipated, the TML-based approach integrates some basic theoretical assumptions, that are well documented in neuroscience (particularly the relationship between preferred music and potential therapeutic effects) with the idea that musical structures and parameters also play an important role in determining therapeutic effects. This means giving specificity to musical proposals by-passing the idea that musical pleasure/pleasure alone can determine any therapeutic effect. A generic factor leading to oversimplification would be that any music you like can have a therapeutic effect.

In order to study music more closely, we need to isolate those characteristics whose variations can potentially generate very different effects. Identifying these parameters is extremely challenging since perception of music differs from the sum of individual musical factors, which are impossible to separate in their interaction (Raglio, 2020). However, it is considered of fundamental importance to connote and classify musical pieces not only from a subjective point of view, but also based on their structural or parametric characteristics. This allows music to be more appropriately related to the effects produced by giving therapeutic interventions greater specificity. Using music listening as a therapeutic tool, Martin-Saavedra et al. (2018) identified 85 studies, and only half of them mentioned at least one musical parameter in their descriptions of interventions. Several studies have attempted to study the relationship between musical parameters and emotions with possible applications in music therapy. Music psychology has, in fact, made an important contribution in this direction (Scherer, 1995; Snowdon et al., 2019), investigating the components and genres that characterize listeners’ musical preferences in relation to personality traits and cognitive styles (Vuoskoski and Eerola, 2011, 2017; Dobrota and Reić Ercegovac, 2015; Greenberg et al., 2015; Eerola et al., 2016; Anderson et al., 2021). A recent review by Reybrouck et al. (2021) shows how the emotional and cognitive processes involved in music listening also depend on the acoustic characteristics of music to the point of hypothesizing possible mathematical functions that could explain the relationship between the sound characteristics of music and the responses evoked in the listener. However, there are many confounding factors such as intrapersonal, interpersonal, and external factors that can significantly influence the actual outcome resulting from listening.

### 5.2. Future perspectives

The approach presented is intended to be an attempt to combine the subjective perspective of music listening with a scientifically oriented approach to determine a link between musical stimulus and therapeutic effect. The data that emerged from the studies provide a fundamental starting point for future investigation related to music listening aimed at treatment. Such an approach could ensure a kind of “modeling” of musical listening also characterized by quantitative implications (thus more objectifiable and standardizable) with respect to different clinical problems. In order to accomplish this aim, one could also apply music-therapeutically oriented music feature extraction techniques, which will allow therapeutic repertoires to be developed based on quantitatively defined musical features. With the support of Artificial Intelligence, one could consequently identify songs with similar characteristics given by the presence of the same musical parameters that can be easily identified and quantified. Recent data processing techniques attributable to Machine Learning and artificial neural network can also define target populations in relation to specific music characteristics/structures and discover the hidden relationships among the considered study’s variables (Raglio et al., 2020b, 2021b). Another interesting aspect of such a therapeutic approach is that it can be offered remotely as Telemedicine intervention (Raglio, 2020) further simplifying the application proposal and reaching potential users more easily. A perspective which can potentially involve many patients/clients with interesting possibilities for development in numerous clinical areas.

I believe that the future of music therapy must consider these multidisciplinary perspectives, trying to combine the music perspective with the scientific perspective, with the aim of consolidating clinical data found in clinical practice.

Finally, what is manualized in this article, because it is thought in the context of Western culture, should be studied when applied in other cultural contexts, considering the variables related to different listening habits (different identities and different musical structures/features). In particular, if the cultural aspect can be considered on the anamnestic level, understanding the impact of musical structures and parameters in relation to their potential effect on the individual with different cultural background could be an important point to consider in TML interventions.

## Data availability statement

The original contributions presented in this study are included in this article/Supplementary material, further inquiries can be directed to the corresponding author.

## Author contributions

AR conceived and designed the study, wrote all sections of the manuscript, and approved the submitted version.
