# Supplementary material for: A novel music-based therapeutic approach: the Therapeutic Music Listening
Source: Front Hum Neurosci. 2023 Jul 14;17:1204593. doi: 10.3389/fnhum.2023.1204593 (PMC10375023; doi:10.3389/fnhum.2023.1204593)
Supplement: Supplementary file 1 [file Data_Sheet_1.PDF]

Supplementary Material

A novel music-based therapeutic approach:
the Therapeutic Music Listening

Alfredo Raglio*

*** Correspondence:** Alfredo Raglio: alfredo.raglio@icsmaugeri.it

1 Supplementary Data

QUESTIONNAIRE 1

(personal/clinical data and musical anamnesis, before Therapeutic Music Listening treatment)

GENERAL AND CLINICAL INFORMATION

Surname and Name:

Age:

Education:

Diagnosis:

Reported problems:

Therapeutic Aims:

Musical training:

☐ No ☐ Yes

Musical practice:

☐ No ☐ Yes

Preferred musical genre (max. 3 preferences)

☐ Pop ☐ Rock ☐ Jazz ☐ Blues

☐ Classical ☐ Folk ☐ Electronic Music ☐ Sacred Music

☐ Opera Music ☐ Movie Music ☐ Rap

☐ Ethnic/Traditional ☐ Other: …………………………………………………..

Preferred composers/songwriters/performers and pieces of music (to list 2 pieces or more):

You listen to music:

☐ Never

☐ Some time

☐ Always (daily)

You prefer:

☐ well-known pieces

☐ unknown pieces

☐ rhythmic pieces

☐ melodic pieces

☐ Slow music

☐ Fast music

Preferred musical instruments:

Music, sounds, genres you dislike:

Why do you want a playlist?

How much do you feel you need it (from 0 to 10)?:

Notes:

QUESTIONNAIRE 2

(Periodical feedback: after first music listening, during the treatment - weekly –

and at the end of the treatment)

Appreciation (0-10):

Effectiveness (0-10):

Pieces to replace:

Raisons:

Other remarks:

Do you feel you need to continue the treatment?

☐ No ☐ Yes

If yes, how much do you feel you need to continue the treatment (0-10)?:

Notes:

2. Supplementary Figures

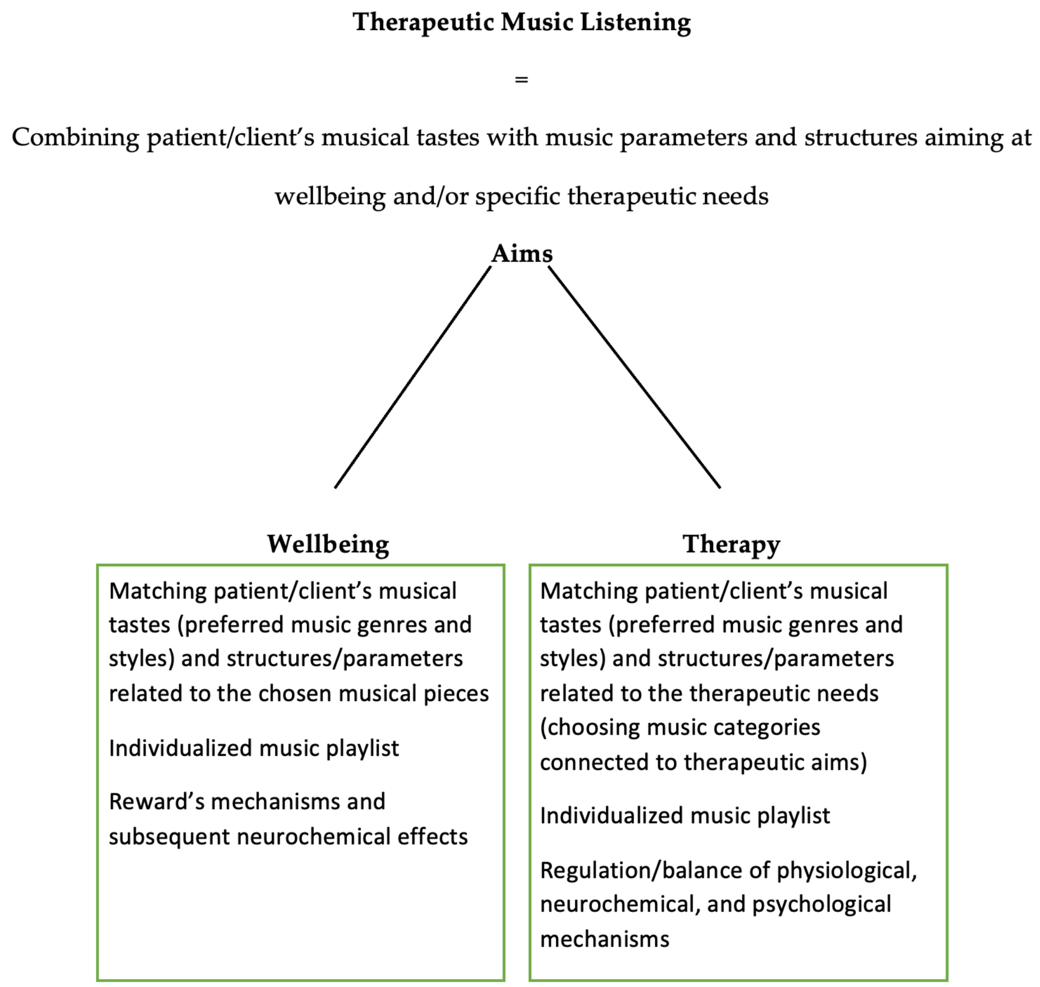

Supplementary Figure 1. Therapeutic Music Listening definition, aims and mechanisms.
